# Prevalence of GSTM1 and GSTT1 null polymorphisms in an admixed healthy Venezuelan population: implications for pharmacogenetic baselines

**DOI:** 10.3389/fgene.2026.1792974

**Published:** 2026-04-23

**Authors:** Mercedes Fernández-Mestre, Eva Salazar-Alcalá, Juan Bautista De Sanctis, Dolores Moreno, Jenny Valentina Garmendia, Oriana Regalado-Gutiérrez, María Johanna Peña

**Affiliations:** 1 Sección Inmunogenética, Laboratorio de Fisiopatología, Centro de Medicina Experimental, Instituto Venezolano de Investigaciones Científicas, San Antonio de los Altos, Venezuela; 2 Instituto de Inmunología Dr. Nicolás E. Bianco, Facultad de Medicina, Universidad Central de Venezuela, Caracas, Venezuela; 3 Institute of Molecular and Translational Medicine, Faculty of Medicine and Dentistry, Palacky University, Olomouc, Czechia; 4 Cátedra de Patología General y Fisiopatología, Escuela Dr. Luis Razetti, Instituto de Medicina Experimental, Facultad de Medicina, Universidad Central de Venezuela, Caracas, Venezuela; 5 Instituto de Investigaciones Biomédicas y de Vacunas Ciudad Bolivar, Caracas, Venezuela

**Keywords:** admix population, detoxification, ethnographic, glutathione S-transferase mu 1, glutathione S-transferase theta 1

## Abstract

**Introduction:**

The Glutathione S-Transferase (GST) family consists of enzymes with widely studied genetic polymorphisms. Current documentation of GST variant distribution across Venezuelan regions is fragmented. This study aims to determine the prevalence of GSTM1 and GSTT1 null genotypes in a healthy urban Venezuelan group and to compare these frequencies with regional and global reference data.

**Methods:**

A cross-sectional descriptive study was conducted on 300 healthy unrelated individuals. Genotyping was performed via multiplex PCR, and frequencies were calculated based on the presence or absence of specific amplicons.

**Results:**

The frequencies of the GSTM1 and GSTT1 null genotypes were 38.67% and 32.67%, respectively. The “double null” genotype was observed in 6.00% of the sample, representing a relevant ethnogeographic heterogeneity.

**Discussion:**

Comparative analysis revealed a divergence from reported data for ancestral Amerindian groups and an allelic distribution pattern reflecting a tri-hybrid genetic architecture intermediate between West African and Southern European references. These findings establish an updated genetic baseline for this urban cohort, highlighting a distinct genotypic distribution within the Venezuelan population. This study underscores the degree of population stratification in the region and provides a descriptive framework for future toxicogenomic research and personalized medicine applications.

## 1 Introduction

The genetic architecture of Latin American populations results from a centuries-long process of admixture involving Amerindian, European, and African lineages. Among the markers used to characterize this genomic diversity, the *GSTM1* and *GSTT1* loci (located on chromosome 1p13.3) are of particular interest due to their common gene deletion polymorphisms, known as “null” genotypes. These variants result in a complete loss of enzymatic activity, potentially significantly altering the Phase II metabolism of specific xenobiotics or drugs. Although these enzymes exhibit marked geographic and ethnic heterogeneity (Garte et al., 2001), their importance in current genomic research lies in their utility as markers of population stratification and gene flow.

Previous studies in Venezuela (Chiurillo et al., 2013) have documented functional conservation (near absence of the deletion) of the *GSTT1* locus in ancestral Amerindian groups. This contrasts with the allelic enrichment of null variants reported in urban areas, suggesting that the distribution of these markers is a sensitive indicator of historical admixture. Notable geographic heterogeneity in the frequencies of *GSTM1* and *GSTT1* deletions is evident across global populations. Within Caucasian groups, for instance, Great Britain has a significantly higher frequency of *GSTM1* deletion, whereas Scandinavian countries exhibit a lower frequency of *GSTT1* deletion. Similarly, among Asians, Japanese subjects show lower deletion frequencies for both genes compared to other regional groups (Garte et al., 2001). In admixed Latin American populations, the frequency of these deletions shows significant variability across countries (Hatagima et al., 2000; Palma-Cano et al., 2017).

However, the degree of internal stratification and the influence of non-native gene flow on the contemporary urban genome require updated characterization. Within the global context of genomic underrepresentation, the Latin American admixture cline requires constant monitoring, as regional genetic markers are not static but evolve through complex demographic transitions. This study is strictly descriptive and population-based; it aims to establish the current prevalence of *GSTM1* and *GSTT1* null genotypes in an urban Venezuelan cohort and to quantify their relationship with ancestral and global references through genetic distance analysis. Consequently, this research intends to establish a redefined genetic baseline, providing a necessary framework for future demographic, evolutionary, and toxicogenomic research in the region.

The Glutathione S-transferase (GST) superfamily consists of enzymes involved in the Phase II metabolism of various compounds. While these enzymes have been extensively studied in the context of xenobiotic biotransformation, their importance in current genomic research lies in their utility as markers of population stratification and gene flow. Previous studies in Venezuela (Chiurillo et al., 2013) have documented a near-absence of the *GSTT1* deletion in ancestral Amerindian groups, in contrast to the higher frequencies reported in urban areas. This suggests that the distribution of these markers is a sensitive indicator of historical admixture. WaConsequently, this research intends to establish a redefined genetic baseline, providing a necessary framework for future demographic and evolutionary research in the region.

## 2 Methods

### 2.1 Population

A cohort of 300 individuals was prospectively enrolled. To ensure a robust benchmark dataset, “apparently healthy” status was defined as the absence of chronic or acute clinical symptoms at the time of recruitment, confirmed by a standardized health questionnaire. All participants were seronegative for various infectious diseases endemic to Venezuela (including HIV, Hepatitis B and C, and Chagas disease) and had no history of autoimmune or oncological diseases. Participants were recruited from the Instituto de Inmunología de la Universidad Central de Venezuela and the Centro de Medicina Experimental del Instituto Venezolano de Investigaciones Científicas. Peripheral venous blood (5 mL) was collected from each participant and stored at 4 °C in EDTA tubes for DNA extraction. Written informed consent was obtained from all participants before sample collection, and the Institutional Ethics Committee approved the study (code 20052308).

### 2.2 Detection of *GSTM1* and *GSTT1* genes

A multiplex PCR protocol was used to determine the presence or absence of the *GSTM1* and *GSTT1* genes, following a modified version of the method described by Lin et al. (1998) and utilizing the specific primers reported by Chen et al. (1996). The reaction mixture consisted of 1X Buffer, 0.75 μM of each primer for the *GSTM1* and *GSTT1* genes, 0.2 μM of β-globin primers (used as an internal control), 2.5 mM KCl, 0.4 mM Tris-HCl, 0.2 mM dNTPs, 3.3 mM MgCl2, and 1.25 U/mL Platinum Taq DNA Polymerase (Invitrogen). For a total reaction volume of 20 μL, 5 μL of genomic DNA (20 μg/mL) was used as a template.

Amplification was performed with an initial denaturation step at 95 °C for 2 min, followed by 30 cycles of denaturation at 94 °C for 1 min, annealing at 64 °C for 1 min, and extension at 72 °C for 1 min. A final extension step was performed at 72 °C for 5 min. The amplified products were visualized via electrophoresis on a 2% agarose gel stained with ethidium bromide at 100 V for 35 min. Genotypes were identified by the presence (functional) or absence (null) of the corresponding bands: 480 bp for *GSTT1*, 215 bp for *GSTM1*, and 268 bp for the β-globin internal control.

Supplementary Figure S1 illustrates the analysis of the assay.

### 2.3 Statistical and comparative analysis

Genotypic frequencies for *GSTM1* and *GSTT1*, as well as their combinations, were determined by direct counting. To ensure technical precision in the Hardy-Weinberg Equilibrium (HWE) calculations, allelic frequencies were estimated based on the equation *p* ^2^ + 2*pq* + *q* ^2^ = 1. Since conventional PCR cannot distinguish between homozygous functional (+/+) and heterozygous (+/−) individuals, the frequency of the null allele (*q*) was derived as the square root of the observed null genotype frequency (*q* = √null frequency), and the functional allele frequency (*p*) was derived as 1 - *q* (Cotton et al., 2000). This approach represents the established methodological standard for analyzing null-allele genetic markers (Falconer and Mackay, 1996).

To contextualize the findings, observed frequencies were compared with data from diverse ethnic groups using Chi-square (*X* ^2^) or Fisher’s exact tests. Furthermore, Nei’s standard genetic distance (*D*) was calculated as *D* = -ln(I), where I represents genetic identity. While future research may examine how epigenetic factors, such as DNA methylation, influence these “molecular phenotypes,” this study remains focused on the foundational genetic distribution. A p-value <0.05 was considered statistically significant.

Supplementary Table S1 illustrates the genotypic and allelic frequencies.

## 3 Results

### 3.1 Estimation of Hardy-Weinberg Equilibrium (HWE)

The distribution of *GSTM1* and *GSTT1* genotypes and the corresponding estimated allelic frequencies for the Venezuelan cohort (n = 300) are summarized in Table 1. The observed null genotype frequency was 0.3867 for *GSTM1* and 0.3267 for *GSTT1*. Under the HWE assumption, the estimated frequency of the null allele (q) was 0.6218 and 0.5715, respectively.

**TABLE 1:** Genotypic and allelic frequencies for *GSTM1* and *GSTT1* (N = 300).

Locus	Genotype (phenotype)	Observed (n)	Observed frequency	Estimated allele frequency
*GSTM1*	Positive (+/+ or +/−)	184	0.6133	p (+): 0.3782
	Null (−/−)	116	0.3867	q (−): 0.6218
*GSTT1*	Positive (+/+ or +/−)	202	0.6733	p (+): 0.4285
	Null (−/−)	98	0.3267	q (−): 0.5715

+: positive, -: null.

### 3.2 Frequency of *GSTM1* and *GSTT1* genotypes and their combinations

The prevalence of the *GSTM1* null genotype was 38.67%, while the *GSTT1* null genotype was observed in 32.67% of the total sample. When analyzing the distribution of genotype combinations, the most frequent profile was the double-positive (*GSTM1*+/*GSTT1*+) at 34.67%, followed by the *GSTM1* null/*GSTT1*+ combination (32.67%). The “double null” genotype (*GSTM1* null/*GSTT1* null) was observed in 6.00% of individuals (Table 2).

**TABLE 2:** Frequency of *GSTM1* and *GSTT1* genotypes and their combinations (n = 300).

Genotype	n = 300	Frequency (%)
*GSTM1* positive	184	61.33
*GSTM1* null	116	38.67
*GSTT1* positive	202	67.33
*GSTT1* null	98	32.67
Genotypic combination		
*GSTM1*+/*GSTT1*+	104	34.67
*GSTM1*+/*GSTT1* null	80	26.67
*GSTM1* null/*GSTT1*+	98	32.67
*GSTM1* null/*GSTT1* null	18	6.00

Supplementary Table S1 contains the complete information on genotypic and allele frequencies.

### 3.3 Comparative global distribution of *GSTM1* and *GSTT1* null genotype frequencies

The frequencies of the *GSTM1* and *GSTT1* null genotypes in our study population, along with a comprehensive comparison with other global populations, are summarized in Table 3.

**TABLE 3:** Comparative global distribution of *GSTM1* and *GSTT1* null genotype frequencies. (Data for global populations adapted and modified from Saitou and Ishida (Saitou and Ishida, 2015); references for individual studies are maintained as cited in the original meta-analysis).

Continent	Population (Location/Group)	n	*GSTM1* null	*GSTT1* null	References
Americas	Current study (Venezuela)	300	0.387	0.327	Current study
	Urban/admixed (Venezuela)	120	0.510	0.110	Chiurillo et al. (2013)
	Bari (ethnic group, Venezuela)	35	0.543	0.114	Chiurillo et al. (2013)
	Panare (ethnic group, Venezuela)	46	0.152	0.065	Chiurillo et al. (2013)
	Pemon (ethnic group, Venezuela)	40	0.400	0.000	Chiurillo et al. (2013)
	Warao (ethnic group, Venezuela)	29	0.517	0.000	Chiurillo et al. (2013)
	Wayuu (ethnic group, Venezuela)	38	0.447	0.079	Chiurillo et al. (2013)
	Colombia	323	0.495	0.517	Martínez Trujillo (2021)
	Mexico (Northwest)	211	0.440	0.110	Palma-Cano et al. (2017)
	Mexico (Northeast)	118	0.480	0.130	Jaramillo-Rangel et al. (2015)
	Mexico (West)	125	0.430	0.030	Gutiérrez-Amavizca et al. (2013)
	Mexico (central)	529	0.330	0.120	Pérez-Morales et al. (2008)
	Mexico (Southeast)	82	0.220	0.170	Sánchez-Guerra et al. (2012)
	Amerindians (Tarahumara, Mex)	-	0.507	0.107	Palma-Cano et al. (2017)
	Mestizos (Mexico)	-	0.442	0.116	Pérez-Morales et al. (2008)
	Brazil (São Paulo/Bahía)	594	0.439	0.231	Gattás et al. (2004)
	White (São Paulo)	233	0.554	0.223	Gattás et al. (2004)
	Black (São Paulo)	137	0.328	0.263	Gattás et al. (2004)
	Mulatto (Bahía)	89	0.360	0.191	Gattás et al. (2004)
	Guaraní (Brazil)	51	0.040	0.120	Gaspar et al. (2002)
	Ache (Paraguay)	67	0.360	0.180	Gaspar et al. (2002)
Africa	Ibo (Abuja)	101	0.23	0.36	Ebeshi et al. (2011)
	Hausa (Abuja)	98	0.37	0.42	Ebeshi et al. (2011)
	Ethiopian (Addis Ababa)	153	0.44	0.37	Piacentini et al. (2011)
	Egyptian (Cairo)	200	0.56	0.30	Hamdy et al. (2003)
	Mandinka (Gambia)	114	0.28	0.40	Kirk et al. (2005)
	Fula (Gambia)	77	0.23	0.47	Kirk et al. (2005)
	Wollof (Gambia)	50	0.16	0.50	Kirk et al. (2005)
	Yoruba (Abuja)	101	0.31	0.35	Ebeshi et al. (2011)
	Sudanese (Khartoum)	114	0.39	0.38	Tiemersma et al. (2001)
	Tunisian (Mahdia)	182	0.54	0.29	Lakhdar et al. (2010)
	Somali (Mogadishu)	100	0.40	0.44	Buchard et al. (2007)
	Ovambo (Windhoek)	134	0.11	0.36	Fujihara et al. (2009)
	Cameroonian (Yaoundé)	126	0.28	0.47	Piacentini et al. (2011)
	Tunisians (Sousse)	186	0.63	0.37	Salem et al. (2011)
Asia	Bahraini (Manama)	167	0.50	0.29	Salem et al. (2011)
	Thai (Bangkok)	320	0.60	0.38	Pakakasama et al. (2005)
	Lebanese (Beirut)	141	0.53	0.38	Salem et al. (2011)
​	Chinese (Beijing)	481	0.44	0.20	Li et al. (2012)
	Indian (Mumbai)	82	0.17	0.22	Nair et al. (1999)
	Chinese (Chengdu)	410	0.51	0.49	Jing et al. (2012)
	Indian (Delhi)	309	0.21	0.27	Singh et al. (2009)
	Chinese (Guangzhou)	412	0.47	0.48	Zhang et al. (2011)
	Vietnamese (Ha Nam)	100	0.42	0.30	Agusa et al. (2010)
	Chinese (Harbin)	226	0.46	0.49	Lu et al. (2011)
	Han (Henan)	212	0.51	0.50	Song et al. (2009)
	Pakistani (Islamabad)	162	0.36	0.10	Khan et al. (2010)
	Indonesian (Jakarta)	162	0.56	0.41	Amtha et al. (2009)
	Druze	159	0.60	0.07	Karban et al. (2011)
	Non-Ashkenazi Jews	172	0.55	0.22	Karban et al. (2011)
	Muslim Arab	101	0.56	0.22	Karban et al. (2011)
	Ashkenazi Jews	96	0.55	0.26	Karban et al. (2011)
	Chinese (Yangzhong)	419	0.51	0.45	Setiawan et al. (2000)
	Kabul, Pashtuns	257	0.42	0.07	Saify et al. (2012)
	Kabul, Tajiks	217	0.48	0.25	Saify et al. (2012)
	Kabul, Hazaras	120	0.53	0.25	Saify et al. (2012)
	Kabul, Uzbeks	62	0.40	0.29	Saify et al. (2012)
	Kashmiri (Srinagar)	195	0.42	0.25	Malik et al. (2010)
	Indian (Kerala)	146	0.27	0.09	Sreeja et al. (2005)
	Thai (Khon Kaen)	94	0.60	0.40	Settheetham-Ishida et al. (2009)
	Japanese (Kitakyushu)	126	0.44	0.44	Katoh et al. (1996)
	Tibetan (Lhasa)	86	0.61	0.36	Yan et al. (2006)
	Filipino (Quezon)	127	0.59	0.25	Baclig et al. (2012)
	Chinese (Meizhou)	512	0.62	0.48	Pan et al. (2011)
	Japanese (Nagoya)	320	0.58	0.43	Niwa et al. (2005)
	Chinese (Qingdao)	366	0.43	0.49	Jiang et al. (2011)
	Saudi (Riyadh)	513	0.55	0.25	Al-Dayel et al. (2008)
	Korean (Seoul)	549	0.51	0.53	Uhm et al. (2007)
	Iranian (Shiraz)	169	0.51	0.21	Moasser et al. (2012)
	Iranian (Tehran)	336	0.28	0.21	Safarinejad et al. (2011)
	Japanese (Tokyo)	203	0.50	0.51	Tamaki et al. (2011)
	Mongol (Ulan Bator)	207	0.46	0.26	Fujihara et al. (2009)
	Han (Wenzhou)	152	0.48	0.49	Chen et al. (2012)
	Han (Xi’an)	763	0.52	0.39	Liu et al. (2009)
	Turkish (Ankara)	231	0.54	0.19	Ada et al. (2012)
Europe	Greek (Athens)	171	0.52	0.10	Dialyna et al. (2001)
	German (Heidelberg)	1251	0.51	0.17	Timofeeva et al. (2010)
	Mediterranean (Barcelona)	192	0.49	0.19	To-Figueras et al. (1997)
	Norman (Basse-Normandie)	120	0.49	0.26	Abbas et al. (2004)
	Danish (Copenhagen)	200	0.53	0.14	Buchard et al. (2007)
	Portuguese (Central-Eastern)	102	0.40	0.18	Ramalhinho et al. (2011)
	Portuguese	121	0.372	0.198	Ramalhinho et al. (2012)
	Scottish (Aberdeen)	383	0.58	0.17	Little et al. (2006)
	Finnish Caucasian (Helsinki)	478	0.42	0.13	Mitrunen et al. (2001)
	Ukrainian (Kiev)	253	0.51	0.14	Ebrahimi et al. (2004)
	Slovenian (Ljubljana)	116	0.54	0.24	Dolzan et al. (2006)
	Polish (Lodz)	233	0.48	0.16	Kargas et al. (2003)
	Spanish (Madrid)	94	0.55	0.28	Piacentini et al. (2011)
	Spanish (Murcia)	173	​	0.179	Muro-Perez et al. (2023)
	Slovakian (Martin)	220	0.48	0.21	Matakova et al. (2009)
	Czech (Brno)	331	0.50	0.22	Holla et al. (2006)
	Norwegian (Oslo)	357	0.51	0.19	Skjelbred et al. (2011)
	Icelandic (Reykjavik)	395	0.54	0.21	Gudmundsdottir et al. (2001)
	Italian (Rome)	143	0.53	0.33	Piacentini et al. (2011)
	Italian (Florence)	546	0.50	0.17	Palli et al. (2005)
	Austrians (Vienna)	305	0.55	0.17	Gundacker et al. (2009)

### 3.4 Internal variability in the venezuelan population

Our findings reveal a significant shift in the genetic landscape of the Venezuelan population concerning Phase II xenobiotic-metabolizing enzymes, highlighting a high degree of previously underestimated inter-population stratification. Statistical analysis confirmed that the null genotype frequencies in the present cohort differ significantly from the 2013 Venezuelan reference data (Chiurillo et al., 2013).

Regarding *GSTM1*, our reported frequency (0.387) is notably lower than the 0.510 previously reported for urban admixed populations (*X* ^*2*^ = 5.37, *p* = 0.020). Notably, comparison with indigenous ethnicities showed no significant divergence from the Wayuu (0.447; *p* = 0.483), Pemon (0.400; *p* = 0.876), or Warao (0.517; *p* = 0.184) groups, indicating a relative conservation of the *GSTM1* locus across these different ethnic and urban strata.

Significantly (*p* < 0.0001), the *GSTT1* null frequency (0.327) in this study is nearly three times higher than the previous national record for an admixed group (0.110). This value far exceeds the frequencies found in ancestral Amerindian lineages, such as the Pemon (0.000; *X* ^*2*^ = 17.58, p < 0.0001) and Wayuu (0.079; *X* ^*2*^ = 10.15, p = 0.001). This sharp discrepancy reflects “within-country variation” driven by local ancestral differences, in which the urban cohort shows allelic enrichment of the null variant that is largely absent in isolated indigenous groups.

### 3.5 Genetic distance with venezuelan subpopulations

The genetic distance analysis reveals a complex pattern of differentiation within the national territory. Using the null allele frequencies (*q*) of *GSTM1* and *GSTT1*, we quantified the divergence between the current cohort and the groups described by Chiurillo et al. (2013). As shown in Table 4, the current urban cohort exhibits its closest overall affinity to the Wayuu (Average *D* = 0.0438), primarily driven by the stability of the *GSTM1* locus (*D* = 0.0035).

**TABLE 4:** Nei’s genetic distance (*D*) between current study and venezuelan reference groups.

Population pair	*D* (*GSTM1*)	*D* (*GSTT1*)	Average *D*	Interpretation
Current study vs. Wayuu	0.0035	0.0841	0.0438	Closest overall indigenous affinity
Current study vs. Pemon	0.0001	0.1758	0.0880	High similarity in *GSTM1* only
Current study vs. Urban/Admixed (2013)	0.0154	0.1248	0.0701	Significant divergence in *GSTT1*
Current study vs. Bari	0.0243	0.1189	0.0716	Moderate divergence
Current study vs. Warao	0.0169	0.3867	0.2018	Highest divergence (fixed *GSTT1*)
Current study vs. Panare	0.0549	0.1343	0.0946	Moderate-High divergence

Conversely, the highest divergence was observed in comparison to the Warao (Average *D* = 0.2018), where the functional fixation of the *GSTT1* allele in the indigenous group contrasts sharply with the urban frequency. The divergence from the 2013 Urban/Admixed reference (*D* = 0.0701) is largely attributed to the *GSTT1* null frequency (*D* = 0.1248$), reinforcing the need for this updated characterization of the contemporary urban genome.

### 3.6 Regional context: the American continent

Compared with neighboring populations, the Venezuelan profile observed in this study shows distinct divergence, highlighting the region’s genetic heterogeneity.

A primary finding is the significant difference between the current Venezuelan cohort and the Colombian population (Martínez Trujillo, 2021). Colombian frequencies for both null genotypes are substantially higher than those found in our sample. Statistical comparison confirms this divergence for *GSTM1* (0.495 vs. 0.387; *X* ^*2*^ = 7.01, p = 0.008) and is even more pronounced for *GSTT1* (0.517 vs. 0.327; *X* ^*2*^ = 22.29, p < 0.0001).

The *GSTM1* (0.387) frequency observed here is comparable to that in central Mexican cohorts (0.330; *X* ^*2*^ = 2.76, p = 0.096), indicating no statistically significant difference and suggesting a similar prevalence of this marker. However, the *GSTT1* (0.327) frequency in our study is significantly higher than that reported for Mexican Mestizos (0.116; *X* ^*2*^ = 44.82, p < 0.0001). Regarding Brazil, our results show a closer affinity to the general population of São Paulo/Bahia (*GSTM1*: 0.439, p = 0.141; *GSTT1*: 0.231, p = 0.003). Notably, the frequency of *GSTT1* (0.327) shows the strongest correlation with the Black population of São Paulo (0.263; *X* ^*2*^ *= 1.98, p = 0.159), for which* no significant difference was found. This affinity likely reflects the substantial African ancestral component in the Venezuelan “mestizaje” (admixture), as high frequencies of GSTT1 null alleles are a well-documented hallmark of several Sub-Saharan African groups.

### 3.7 Global distribution and ethnogeographic implications

To evaluate the relationship between the healthy Venezuelan population and global cohorts, Nei’s Genetic Distance (*D*) was calculated using the null allele frequencies (*q*) derived from global data (Table 5).

**TABLE 5:** Nei’s genetic distance (*D*) between venezuela and reference populations.

Population pair	*D* (*GSTM1*)	*D* (*GSTT1*)	Average *D*	Interpretation
Venezuela vs. Portugal	0.0003	0.0452	0.0227	High affinity to southern Europe
Venezuela vs. Ibo (Nigeria)	0.0812	0.0031	0.0421	Strong African affinity (*GSTT1*)
Venezuela vs. Spain (Murcia)	--	0.0614	--	Divergence from Iberian baseline
Venezuela vs. Mexico (central)	0.0125	0.1684	0.0904	Moderate regional divergence
Venezuela vs. China (Beijing)	0.0121	0.2312	0.1216	High divergence

On a global scale, our *GSTT1* null findings (0.327) are highly consistent with frequencies typically observed in West African populations, such as the Ibo of Nigeria (0.36; *X* ^*2*^ = 0.35, *p* = 0.552). The lack of statistical significance in this comparison suggests a robust genetic affinity, reinforcing the historical impact of trans-Atlantic gene flow on the current Venezuelan genetic architecture.

In stark contrast, comparisons with Southern European cohorts reveal a highly significant divergence. Our finding of a *GSTT1* null frequency of 0.327 differs substantially from recent reports in the Iberian Peninsula, such as Muro-Perez et al. (2023), who described a frequency of 0.179 in the population of Murcia, Spain (*X* ^2^ = 14.82, *p* = 0.0001). This significant gap between our cohort and modern Spanish samples—including the Portuguese frequency (0.198; *p* = 0.008) and the Italian sample (0.17; *p* = 0.0001)—underscores that the Venezuelan genetic landscape is not merely a reflection of its European ancestry. Instead, the higher frequency of the null allele in our study group suggests a robust contribution from other ancestral sources, likely Sub-Saharan African lineages, which are known to possess higher frequencies of the *GSTT1* deletion.

Interestingly, the Venezuelan *GSTM1* null frequency (0.387) is notably lower than the frequencies found in the European populations that historically migrated to the region. Comparison with Spanish (0.55; *X* ^2^ = 7.73, *p* = 0.005$) and Italian (0.53; *X* ^2^ = 8.13, *p* = 0.004) cohorts reveals a significant divergence, as these groups exhibit a much higher prevalence of the deletion. However, our sample aligns closely with the Portuguese frequency reported by Ramalhinho et al. (2012) (0.372; *X* ^2^ = 0.076, *p* = 0.782), showing no statistically significant difference. This suggests that the Mediterranean ancestral contribution to the Venezuelan *GSTM1* locus may be more closely associated with specific Southern European lineages, or potentially reflects a balancing effect from Amerindian ancestral components where the functional gene is more prevalent.

In contrast to the European-African axis, many Asian populations exhibit significantly higher frequencies of the *GSTT1* null genotype than observed in our Venezuelan sample (e.g., Korea: 0.53; *X* ^2^ = 32.89, p < 0.0001). Similarly, our *GSTM1* frequency remains lower than in many East Asian populations (e.g., Thailand: 0.60; p < 0.0001).

Overall, the Venezuelan population occupies a unique intermediate position between European and African coordinates. While the *GSTM1* axis shows a shift toward Southern European clusters (e.g., Portugal), the *GSTT1* axis reveals a robust contribution from Sub-Saharan lineages. This genetic signature differentiates the Venezuelan cohort from both its ancestral precursors and other regional admixed groups, such as the Colombian population, which exhibits higher null frequencies for both loci (*p* < 0.01).

### 3.8 Synthesis of the genetic landscape

**FIGURE 1:**
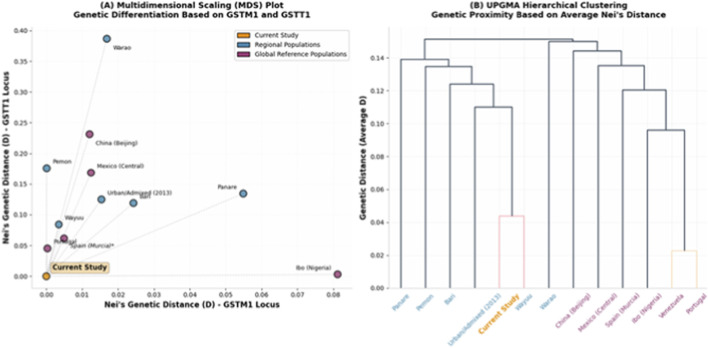
Genetic Landscape and Population Relationships Based on GSTM1 and GSTT1 Null Alleles. **(A)** Multidimensional Scaling (MDS) plot: Illustrates the differentiation of the studied cohort based on Nei’s genetic distance (D). The X-axis primarily reflects genetic distance at the *GSTM1* locus, while the Y-axis captures variability at the *GSTT1* locus. The current study (orange) shows close affinity to Southern European (Portugal) and certain Amerindian (Wayuu) groups at the *GSTM1* locus, while its *GSTT1* profile shifts toward West African references (Ibo, Nigeria). **(B)** UPGMA Hierarchical Clustering: Dendrogram representing genetic proximity based on average Nei’s distance. The urban Venezuelan cohort (orange) clusters closely with contemporary admixed references and the Wayuu group, highlighting a distinct lineage separate from European-only or Asian populations.

The MDS plot (Figure 1A) provides a spatial representation of the genetic landscape for the *GSTM1* and *GSTT1* loci. The Venezuelan population occupies a central position within the multidimensional space, reflecting its multi-ancestral genomic architecture. A high affinity is observed toward the Southern European cluster (specifically Portugal and Italy) along the *GSTM1* axis. Concurrently, a significant shift toward the West African coordinate (Nigeria-Ibo) is evident in the *GSTT1* axis. This displacement is driven by the elevated frequency of the null genotype (32.67%) in the study group, which represents a twofold increase compared to recent Mediterranean reports, such as the 17.9% observed in Murcia, Spain (Muro-Perez et al., 2023).

The UPGMA-based dendrogram (Figure 1B) further clarifies these relationships through hierarchical branching. The studied cohort is positioned within a primary cluster that includes Southern European references (Portugal and Spain-Murcia). However, this cluster converges at a secondary level with a node composed of Sub-Saharan African populations (Nigeria-Ibo and Nigeria-Yoruba). This topology confirms a dual European-African genetic convergence in the Venezuelan sample. In contrast, the branches representing Asian (China and Japan) and Amerindian-influenced (Central Mexico) populations exhibit the highest genetic distances (D > 0.12), forming distinct outgroups relative to the Venezuelan cohort.

## 4 Discussion

The present study provides a comprehensive update on the distribution of *GSTM1* and *GSTT1* null polymorphisms in a healthy Venezuelan urban cohort. Our results reveal a *GSTM1* null frequency of 38.67% and a *GSTT1* null frequency of 32.67%, uncovering a genetic structure that diverges significantly from previous national benchmarks. This shift highlights the complex inter-population stratification within the Venezuelan territory and the impact of non-native gene flow on the contemporary urban genome.

The observed *GSTM1* null frequency (38.67%) is lower than the typical prevalence reported for European (50%–55%) and East Asian (50%–60%) populations. Interestingly, this frequency shows no significant divergence from ancestral Amerindian groups such as the Wayuu (44.7%) and Pemon (40.0%), suggesting a relative conservation of the functional *GSTM1* allele across different Venezuelan ethnic strata. This pattern aligns with the lower range of Mediterranean populations, showing a notable proximity to Portuguese references (*D* = 0.0003). This suggests that the *GSTM1* locus has remained relatively stable during the admixture process.

In contrast, the *GSTT1* null frequency (32.67%) represents a nearly three-fold increase compared to the 11.0% previously reported for urban admixed Venezuelans. This high prevalence is the most striking feature of our cohort, as it sharply contrasts with the near-absence of the deletion in indigenous Amerindian lineages, such as the Pemon (0%) and Wayuu (7.9%). The transition from a predominantly functional *GSTT1* ancestral state to a high-frequency null state in the modern urban population serves as a clear genetic marker of substantial historical gene flow.

The calculation of Nei’s genetic distance (*D*) provides a quantitative framework to interpret these shifts. Our cohort exhibits a marked divergence from the Iberian baseline, specifically from Spain-Murcia (*D* = 0.0614 for *GSTT1*), moving instead toward a strong statistical affinity with West African populations (*D* = 0.0031 for *GSTT1* with the Ibo of Nigeria). This contrast underscores the pivotal role of trans-Atlantic gene flow in shaping the modern Venezuelan gene pool.

The dual affinity observed—where the population clusters with Southern European references for *GSTM1* and with African references for *GSTT1*—highlights the tri-hybrid nature of the Venezuelan genome. This “mosaic pattern” suggests that the Venezuelan pharmacogenetic profile is shaped by locus-specific ancestral signatures, where different loci carry distinct ancestral footprints depending on the intensity of gene flow. Such a mosaic architecture is essential for “phenotypic prediction”, as the metabolic capacity of an individual cannot be inferred from a single ancestral axis.

The significant differences between our results and the data from Chiurillo et al. (2013) emphasize the high degree of internal stratification in Venezuela. These discrepancies, driven by demographic dynamism and varying degrees of admixture, argue that a single “national average” for null-allele markers is insufficient for clinical trial design in Venezuela. Relying on a generalized average may overlook the specific needs of stratified subpopulations, potentially leading to suboptimal therapeutic outcomes. Furthermore, the application of “polygenic risk scores” or pharmacogenetic baselines developed primarily in European cohorts may “exacerbate health disparities” in highly admixed populations like ours. Our data underscores that understanding the “tri-hybrid nature” of the Venezuelan genome is not merely an anthropological exercise but a fundamental requirement for the implementation of “personalized and genomic medicine”. By accounting for specific ethnogeographic ancestry at the locus level, we can better predict individual responses to xenobiotics and refine clinical protocols.

In conclusion, the move toward an intermediate genetic position between European and African coordinates confirms that the urban Venezuelan genome is a unique product of historical gene flow. This study emphasizes that genetic markers are not static but evolve through complex demographic transitions, and underscores the necessity of accounting for specific ancestral “mosaic patterns” to ensure the equitable advancement of precision medicine in the region.

## 5 Conclusion

This study provides a comprehensive characterization of *GSTM1* and *GSTT1* null polymorphisms, establishing a redefined genetic baseline for the urban Venezuelan population. Our findings reveal a unique allelic distribution shaped by the convergence of West African and Southern European lineages over an ancestral Amerindian substrate.

The significant enrichment of the *GSTT1* null allele—which stands in sharp contrast to its near-absence in regional indigenous groups—serves as a robust genomic signature of the profound impact of trans-Atlantic gene flow on the contemporary local gene pool. Furthermore, the dual affinity observed through genetic distance analysis confirms that the urban Venezuelan population occupies a distinct position in the global genetic landscape, characterized by a “mosaic” tri-hybrid recombination that differentiates it from both its ancestral precursors and other regional admixed cohorts.

These results underscore the high degree of internal stratification within the national territory and establish a fundamental framework for future studies to ensure they capture the true demographic complexity of the Latin American “mestizaje”. By demonstrating that regional genetic markers are not static but evolve through complex demographic transitions, this research provides a critical reference for the implementation of personalized medicine and pharmacogenetic screening in South American admixed populations.

Moving forward, accounting for specific ethnogeographic ancestry in future pharmacogenetic research is a necessity, not an option. Understanding the interacting roles of genomic and environmental factors is vital for the global application of pharmacogenetics. This study ensures that future clinical and evolutionary research in the region is grounded in a baseline that accurately reflects the ancestral diversity of the contemporary Venezuelan genome, ultimately bridging the gap between population genetics and equitable Precision Medicine.

## Data Availability

The original contributions presented in the study are included in the article/Supplementary Material, further inquiries can be directed to the corresponding authors.
