# Supplementary material for: Prevalence of GSTM1 and GSTT1 null polymorphisms in an admixed healthy Venezuelan population: implications for pharmacogenetic baselines
Source: Front Genet. 2026 Apr 23;17:1792974. doi: 10.3389/fgene.2026.1792974 (PMC13148882; doi:10.3389/fgene.2026.1792974)
Supplement: Supplementary file 1 [file DataSheet1.pdf]

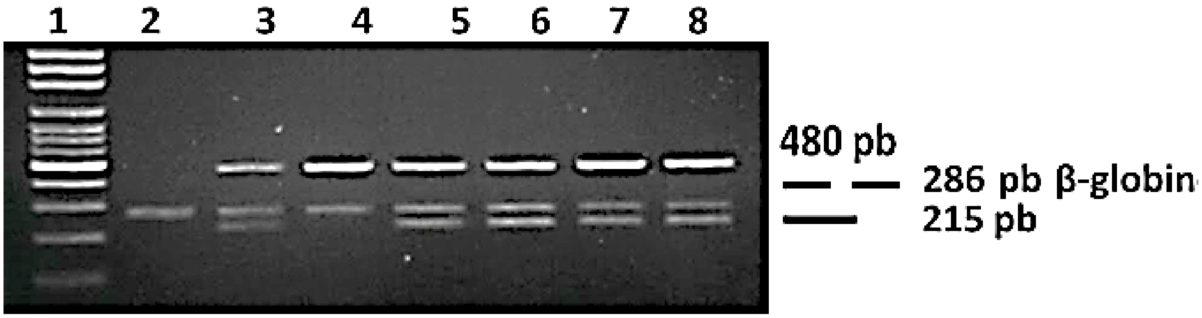

Supplemental Figure 1. Representative electrophoresis gel of multiplex PCR for *GSTM1* and *GSTT1* polymorphisms. Lane 1 shows the 100 bp molecular size marker (Axygen) used to determine the size of the amplified products. Lanes 2 to 8 correspond to individual samples showing bands for *GSTM1* (215 bp), *GSTT1* (480 bp), and β-globin (268 bp) as an internal control. Lane 2 represents a double null individual (*GSTM1-*/*GSTT1-*); lanes 3 and 5 to 8 show individuals positive for both genes (*GSTM1+*/*GSTT1+*); and lane 4 represents an individual with the *GSTM1* null and *GSTT1* present genotype (*GSTM1-*/*GSTT1+*).

Supplemental Table 1. Genotypic and Allelic Frequencies for *GSTM1* and *GSTT1* (N=300)

**Locus**	**Genotype (Phenotype)**	**Observed (n)**	**Observed Frequency**	**Estimated Allele Frequency**
GSTM1	Positive (+/+ or +/-)	184	0.6133	p (+): 0.3782
	Null (-/-)	116	0.3867	q (-): 0.6218
GSTT1	Positive (+/+ or +/-)	202	0.6733	p (+): 0.4285
	Null (-/-)	98	0.3267	q (-): 0.5715

+: positive, -: null
